# The effect of statement type and repetition on deception detection

**DOI:** 10.1186/s41235-019-0194-z

**Published:** 2019-09-23

**Authors:** Daniella K. Cash, Rachel E. Dianiska, Sean M. Lane

**Affiliations:** 1 Louisiana State University, Baton Rouge, LA USA; 2 Iowa State University, Ames, IA USA

## Abstract

**Background:**

Deception is a prevalent component of human interaction. However, meta-analyses suggest that discriminating between truthful and deceptive statements is a very arduous task and accuracy on these judgments is at chance levels. To complicate matters further, individuals tell different types of lies. The current studies examined how an individual’s ability to accurately discriminate between truthful and deceptive statements is affected by the way truths and lies are conveyed. Participants judged the veracity of statements given by speakers who told truths or lies about a performed action by describing that action or denying that it had occurred. Additionally, these statements also differed with regard to how often the lie had been repeated (i.e., practiced), either once or thrice.

**Results:**

The results were largely in line with the prevailing notion that it is difficult to successfully differentiate between truthful and deceptive statements, but also showed that performance was moderated by statement type and repetition. The results revealed that participants were more accurate in discriminating unrepeated descriptions than repeated descriptions, but this difference was not seen for denial statements. Additionally, participants were more likely to believe practiced (repeated) statements, both truthful and deceptive.

**Conclusion:**

The results show that repeated statements as well as shorter denials can increase the difficulty of differentiating truthful from deceptive statements. Additionally, these findings suggest that truthful statements also benefit from repetition with regard to enhancing their believability.

## Significance

The ability to know whether or not someone is lying or telling the truth is a difficult task, and one that people generally perform quite poorly on. To further complicate an already strenuous task, people are not limited in the ways that they can either provide a truthful or deceptive statement. Two types of statements that people can provide are longer, more detailed accounts (referred to here as descriptions) or shorter, less descriptive accounts where they reject an event having occurred (referred to here as a denial). Additionally, both liars and truth tellers may decide (or fail to decide) to practice these accounts. The goal of the current studies was to assess how these different factors may make an already difficult task into one that is even harder. Participants provided veracity judgments for speakers who provided statements regarding whether they did or did not perform an action. These actions were low-stakes lies pertaining to everyday actions (e.g., I did not bounce the ball). The results were in line with earlier research showing that lying is a difficult task and affirmed our hypothesis that the type of lie can also impact this task. Specifically, it was easier for participants to accurately identify unpracticed statements as opposed to practiced statements when the statements were longer descriptions, but there was no difference in accuracy for denial statements. Additionally, practicing a statement made both truthful and deceptive descriptive statements more likely to be believed, which suggests that practice can be beneficial even for truthful individuals.

## Introduction

Deception is a ubiquitous and essential part of human interaction (DePaulo et al., 2003). Despite research showing that individuals lie on average twice a day (DePaulo, Kashy, Kirkendol, Wyer, & Epstein, 1996), meta-analyses reveal that we are poor at detecting deception and only differentiate truthful statements from deceptive statements 54% of the time (Bond & DePaulo, 2006). Furthermore, although there are some studies that have identified specific groups that perform better when making these judgments (i.e., secret service agents and groups with interest in deception detection; Ekman & O’Sullivan, 1991; Ekman, O’Sullivan, & Frank, 1999), these studies have been criticized as being a statistical fluke as opposed to truly representing individuals who are better at discriminating between truthful and deceptive statements (Bond & Uysal, 2007). Additionally, research has shown that individuals who would be expected to have more experience with detecting deception (e.g., law enforcement, judges, psychiatrists, etc.) do not perform any better than novices - on average, 54.51% and 53.31% accurate identification of lies/truths, respectively (Bond & DePaulo, 2006). The fact that our ability to detect lies appears to be only moderately better than a coin flip has prompted numerous questions as to why performance is so poor and what can researchers do to improve it.

To answer this question, researchers have proposed two potential accounts (Vrij, 2008). The first account posits that lie detection is poor because people have an inaccurate representation as to what behavioral components are indicative of deception. Therefore, these errors arise due to incorrect social perceptions about the liar. The second account suggests that it is not the fault of the person for incorrectly interpreting social cues; rather there are limited behavioral cues between liars and truth tellers for people to utilize in their decision-making process. With little to no information to rely on, this account implies that it is the nature of the lie detection task that results in our modest ability to detect deception. Hartwig and Bond (2011) addressed this question through a series of meta-analyses and their findings revealed that the inability to discriminate between truthful and deceptive individuals is due to a lack of behavioral differences between truth-tellers and liars. These findings suggest that it is the latter and not the former explanation for poor deception detection.

### Cues to deception

When attempting to evaluate the veracity of a statement, there are two primary cues that we can rely on: behavioral cues provided by the speaker and the content of the provided account. The emotion framework of deception posits that a liar experiences either fear, guilt, or excitement, resulting in the liar providing behavioral cues indicative of that arousal, such as more speech errors and a higher pitched voice (Ekman, 1992). This notion seems intuitive and when asked to describe what indicators would be present when an individual is trying to deceive another person, it is common to receive answers such as more grooming behaviors (e.g., playing with one’s hair), stories that lack coherence, and marked gaze aversion (Global Deception Research Team, 2006). In fact, a worldwide survey revealed that of the 58 countries that were represented, the majority of respondents in 51 countries thought that gaze aversion was an accurate indicator of guilt (Global Deception Research Team, 2006). Though certain indicators do seem to be weakly related to deception (e.g., voice pitch, pupil size), many commonly suggested indicators are unrelated, such as gaze, blinking, and speech disturbances (DePaulo et al., 2003), highlighting the difficulty of using such cues to differentiate between truthful and deceptive statements. However, relying on behavioral cues is one approach an individual may take when attempting to assess veracity.

Another way to assess statements for veracity is to consider their content, rather than behavioral cues. A cognitive framework proposes that the act of deception is cognitively demanding and this makes it harder for a liar to create a fluid and compelling account (see Vrij, 2015 for a review). A person who is under cognitive load is likely to speak slower, display more speech errors, and make fewer gestures and movements during their account (Ekman & Friesen, 1972; Goldman-Eisler, 1968). The notion that there is a difference in cognitive load is supported by studies that exploit these differences for liars and truth tellers. This includes manipulations that require participants to maintain eye contact with an interviewer (Vrij, Mann, Leal, & Fisher, 2010), complete additional tasks when providing their account (Debey, Verschuere, & Crombez, 2012), providing accounts in reverse chronological order (Evans, Michael, Meissner, & Brandon, 2013; Vrij et al., 2008), and having participants “take turns” when providing their accounts (Vernham, Vrij, Mann, Leal, & Hillman, 2014). These findings suggest that it is also possible to rely on content-based cues when assessing veracity.

While it is unlikely that researchers will identify a single verbal or nonverbal cue that serves as a perfect indication of deception (i.e., Pinocchio’s nose), by better understanding what type of false statement we are likely to incorrectly believe or alternatively, what truthful statements will be deemed deceptive, the current studies explore the impact that different statements can have on deception detection. Specifically, we focus on a statement’s veracity, whether the statement was practiced (repeated) or not, and whether the statement is a description or a denial. Additionally, we also manipulated whether the speaker was referring to an action they performed or one that they observed. Typically, most deception studies ask participants to judge one piece of information per speaker. However, a novel component that the current studies add to the literature is that participants were asked to assess multiple statements provided by the same speaker, allowing participants to become familiar with what the speaker might look like when they are lying or telling the truth. By understanding the finer points of where deception is more or less likely to be successful, researchers can make better informed attempts at trying to improve lie detection rates.

### Veracity and type of statements

Not only is it important to be able to recognize a lie, it is equally important to recognize a truthful statement. To this end, understanding biases that might impact our ability to distinguish between truths and lies is important. One bias that has been demonstrated in the literature is known as the *truth bias* wherein people are usually more accurate when they are asked to judge truthful statements as opposed to deceptive statements; however, this finding stems more from the fact that participants seem to be more inclined to view a statement as truthful as opposed to truly being more accurate (DePaulo, Charlton, Cooper, Lindsay, & Muhlenbruck, 1997; Vrij, 2000). In fact, Vrij and Baxter (1999) found that this truth bias is dependent on the type of statement that was made. Participants watched 20 videos of people telling the truth and 20 videos of people telling a lie. Furthermore, half of these statements were more elaborated in length (i.e., descriptions) or statements with little information (i.e., denials). The results revealed that the longer, descriptive statements elicited a truth bias, where participants were more likely to judge the statement as being truthful. Conversely, the denials were judged to be deceptive more often than they were judged as truthful. A critical difference between Vrij and Baxter (1999) and the current study is that each statement was given by a different person, preventing the participant from being able to look for cues to deceptions within an individual speaker. These findings suggest that not only is there a difference in our ability to discern truthful statements from lies, but the type of statement that is provided impacts these judgments.

### Repetition or practice of statements

Given that it requires more cognitive resources to spontaneously construct a lie than to provide a truthful statement (Vrij et al., 2008), it is intuitive that being able to practice your lie might enhance your believability. Specifically, practicing the lie might reduce some behavioral and content cues that indicate deception, such as being able to provide longer accounts with more details that might be more compelling to a person trying to judge the statement’s veracity. Although they did not directly compare repeated lies to non-repeated lies, Bond Jr and DePaulo (2006) found that when participants knew that they were going to be asked to lie, detectors of deception were less accurate at identifying that the statement was deceptive as opposed to when the participant had to spontaneously generate the lie. In a similar vein, Vrij et al. (2009) found that asking both liars and truth tellers a series of unanticipated questions (e.g., to draw the spatial layout of a location) resulted in liars having more difficulty in providing answers compared to truth tellers. This questioning approach resulted in liars being unprepared to answer these questions, allowing for 80% of liars and truth tellers to be accurately categorized, suggesting that there are techniques that can be beneficial when making veracity assessments.

Research has also demonstrated that simply practicing the act of lying made it easier for people to lie later on (Van Bockstaele et al., 2012). Van Bockstaele et al. (2012) found that participants who were trained to lie rather than tell the truth found it easier to lie than participants who had been trained to tell the truth. However, this difference was only seen for items that had been presented once in the training phase as opposed to novel test items, highlighting that even providing a lie once earlier makes it easier to lie about that item at a later time. Taken as a whole, these studies provide a reason to expect that practicing a lie is indeed beneficial to liars with regard to improving their ability to deceive. However, it is also important to evaluate how practice may or may not impact the believability of truthful statements as truth tellers could potentially be at a disadvantage when their truthful statement is compared to a deceptive and potentially practiced statement.

### Current studies

The current set of studies was designed to test how statement type (i.e., denials or descriptions), veracity (i.e., truth or lie), and repetition (i.e., rehearsed once or rehearsed repeatedly), as well as the role of speaker (i.e., actor or observer), impacts individuals’ ability to detect deception in everyday situations. The video material was taken from a study by (Dianiska, Lane, Viera & Cash: Type of lie differentially influences forgetting and false memory, in preparation) examining the impact of these variables on source memory. The current study is unique in that participants watched four different speakers provide 32 statements that are either truthful or deceptive. We first explored the utility of our novel paradigm in detecting deception with experiment 1, and then expanded our stimulus materials to ensure enough variability amongst speakers to capture real-world variability in experiment 2. Statements were designed to be about simple actions, mimicking how a person may provide a low-stakes lie to people with whom they are familiar (i.e., a *white* lie).

Both experiment 1 and experiment 2 conformed to a 2 (statement type: denial, description) × 2 (veracity: truth, lie) × 2 (repetition: once, thrice) × 2 (role of speaker: actor, observer) mixed design. Statement type, veracity, and repetition were all within- subjects manipulations, allowing participants to be exposed to many different types of lies provided by the same person. Role of speaker was a between-subjects factor in which participants saw a person talking about their own actions (actor) or the actions of another individual (observer). This design allowed us to expand on what is known about lie detection and examine the contributions of different components of a statement that might influence individuals’ ability to discriminate truths from lies. Based on previous research, we expected that it would be more difficult for participants to discriminate between truthful and deceptive denial statements than descriptions. We also expected that repeated description statements (i.e., those that were practiced three times) would be harder to successfully discriminate than statements that had not been repeated. We did not have any specific predictions for the role of speaker manipulation as this was an exploratory factor.

## Methods for experiments 1 and 2

### Participants

A total of 116 participants were recruited from the experimental pool at a large southern university for experiment 1. The mean age in the group was 19.51 years (SD = 2.43). Of these participants, 73.3% identified as being Caucasian and 81% identified as being female. Participants for experiment 2 (*N* = 125) were also recruited from this pool, but participants who had completed experiment 1 were not allowed to take part in experiment 2. The mean age of the group in experiment 2 was 19.78 years (SD = 1.42). Of these participants, 72% identified as being Caucasian and 80% identified as being female. Participants were given course credit in exchange for their time. The number of participants needed for the current study was determined by calculating repeated measures analysis of variance (ANOVA) for a within-between interaction. Using an effect size of *f* = 0.1, this resulted in a required sample of 110 participants for both studies. All conditions are reported and no participants were excluded from the analyses. All American Psychological Association (APA) guidelines were adhered to during this study (Institutional Review Board (IRB) approval code E5167).

### Materials

#### Videos

The current study utilized a set of videos that were obtained from Dianiska et al. (in preparation). These videos contained participants (henceforth referred to as *speakers*, given that they were not the participants used in the current study) making a series of statements about simple actions (e.g., bouncing a ball). The speakers provided statements about actions that they either performed (actors) or observed (observers). They then either told the truth or lied about performing or observing these actions to a video camera. Speakers could lie or tell the truth about an action in two ways: *describing* the action as it had been performed (or observed) or *denying* having performed (or observed) the action. Furthermore, some statements were only provided once, while other statements were repeated three times, giving speakers more practice on some item types than others. Thus, participants in the current study were exposed to eight different statement types from actors and observers, that varied according to veracity (truth, lie), statement type (descriptions, denials), and repetition (once-rehearsed, thrice-rehearsed). For example, a speaker in the actor condition who provided a lie-describe-once statement would describe an action that they did not perform earlier by creating a false description of how they had performed the action.

Each speaker in the study by Dianiska et al. provided a total of 64 statements, resulting in 8 statements of each item type per speaker. The order of what type of statement was provided by the speaker was randomized. The speaker’s video was then edited so that each statement was presented in isolation, resulting in 64 video clips per speaker. Silences where the speaker was reading the prompt or instructions provided by the experimenter were edited out. These videos were then used in the current studies where participants watched the speakers provide their statements and then provided veracity assessments.

Of the 64 statements provided by each speaker in the study of Dianiska et al., participants in the current study viewed only 32 videos. Participants viewed 16 unpracticed (i.e., once-rehearsed) statements, and the final trial of the 16 practiced (i.e., thrice-rehearsed) statements. When items were rehearsed three times, we only showed participants the speaker’s third and thus most-practiced rehearsal. Participants evaluated statements from four unique speakers in each experiment. Thus, participants viewed 128 statements in total, comprising 16 unpracticed statements and 16 practiced statements from four unique speakers.

For experiment 1, four speakers from both the actor condition and the observer condition were randomly selected to be the stimuli for the current study, resulting in a total of eight speaker video sets for the entire experiment. Participants saw either four actor videos or four observer videos. In experiment 2, we used a new sample of eight actor and eight observer videos drawn from (Dianiska, Lane, Viera, and Cash in preparation). This resulted in two sets of four videos for both the actor and observer conditions. Each participant in experiment 2 viewed 4 videos in total. The order that statements were shown was held constant across all participants, in order to assess follow-up questions for each speaker (e.g., “To what degree do you think this person is a believable liar?”). On average, denial statements tended to last approximately 2–4 s while description statements were approximately 10–18 s.

The only difference between experiment 1 and experiment 2 involves the videos that were used. In experiment 1, participants were randomly assigned to see either actor or observer videos (i.e., there were only two sets of videos to which participants could be assigned). In experiment 2, we randomly drew another set of videos, but this time included two versions of each condition (i.e., two actor versions and two observer versions) to which participants could be randomly assigned, resulting in four potential sets of videos. The goal of experiment 2 was to replicate the findings of the first experiment with a broader sample of speakers.

### Procedure

Participants were recruited to take part in the study via an online registration system. Upon arrival, participants were asked to provide informed consent to take part in the study. Participants then read the task instructions that told them they would view a series of video statements and be asked to indicate whether they believed the person to be lying or telling the truth for each statement. Participants were not told how many statements would be deceptive or truthful, only that there would be a mixture of both types of statements. Participants provided a rating on a 6-point Likert scale where “1” indicated that they thought the person was “Definitely lying” and “6” indicated that they thought the person was “Definitely telling the truth.” Each participant saw four different speakers who each provided 32 statements. After participants had seen all of a speaker’s statements, they were also asked to provide judgments about that person’s overall veracity and comfort when lying across all 32 videos (e.g., did this person tell the truth or lie more often? To what degree do you think this person is a believable liar?). Upon viewing all of the videos, participants were asked to indicate what cues they used when assessing whether the person was lying or telling the truth (e.g., how much did you weight the person’s body language when deciding whether they were lying or telling the truth?). Participants were then provided demographic information and were then thanked and debriefed.

## Results

### Experiment 1

For each video response, we collapsed responses across ratings of truthfulness (1–3) or deceptiveness (4–6) ratings to provide a dichotomous outcome of truth or deception. For each response type, we separated the proportion of false statements (i.e., lie deny-once, lie deny-thrice, lie describe-once, and lie describe-thrice) identified as deceptive (hits) and the proportion of truthful statements (i.e., truth deny-once, truth deny-thrice, truth describe-once, and truth describe-thrice) identified as deceptive (false alarms). We then computed signal detection estimates of discrimination accuracy (*d’*) and response bias (*c*) for each response type (i.e., deny once, deny thrice, describe once, describe thrice). High values of *d’* indicate greater ability to distinguish between deceptive and truthful statements. More positive values of *c* indicate a tendency to judge statements as truthful, and more negative values of *c* indicate a tendency to judge statements as deceptive. Although we present the analyzed signal detection measures in text, overall accuracy ratings for all item types in both experiments can be seen in “Appendix”.

#### Discrimination accuracy

We first conducted 2 (role: actor, observer) × 2 (statement type: deny, describe) × 2 (repetition: once, thrice) mixed factorial ANOVA was first conducted on the discrimination accuracy measure, with role serving as the between-subjects variable and statement type and repetition as within-subjects variables. Cell means and standard deviations can be found in Table 1. We observed significant main effects of repetition (*F* (1, 114) = 13.44, *p* < .01, η_p_^2^ = .11) and role (*F* (1, 114) = 4.53, *p* = .04, η_p_^2^ = .04). Discrimination accuracy was significantly worse for statements that had been rehearsed repeatedly compared to statements rehearsed only once (*d* = 0.34, 95% CI [0.15, 0.53]) and for statements where the speaker described actions they had observed compared to statements about actions the speaker had performed themselves (*d* = 0.40, 95% CI [0.03, 0.76]).

**Table 1:** Results in experiment 1

	Hit rate	False Alarm rate	*d’*	*c*
Actor				
Deny once	0.47 (0.21)	0.41 (0.21)	0.17 (0.48)*	0.17 (0.56)
Deny thrice	0.41 (0.21)	0.45 (0.19)	− 0.13 (0.56)	0.21 (0.53)
Describe once	0.56 (0.17)	0.53 (0.18)	0.07 (0.46)	−0.14 (0.44)
Describe thrice	0.50 (0.19)	0.53 (0.20)	−0.10 (0.48)	−0.04 (0.50)
Observer				
Deny once	0.49 (0.14)	0.60 (0.14)	−0.28 (0.45)*	−0.13 (0.33)
Deny thrice	0.44 (0.16)	0.46 (0.19)	−0.06 (0.50)	0.15 (0.50)
Describe once	0.53 (0.20)	0.46 (0.15)	0.19 (0.46)*	0.01 (0.44)
Describe thrice	0.36 (0.13)	0.44 (0.15)	−0.23 (0.42)*	0.28 (0.35)

Standard deviations are in parentheses. Values for *c* greater than 0 indicate a truth bias

*Represents values of *d’* that are significantly different from chance performance

We further observed significant two-way interactions between statement type and role (*F* (1, 114) = 5.02, *p* = .03, η_p_^2^ = .04), as well as between statement type and repetition (*F* (1, 114) = 8.90, *p* < .01, η_p_^2^ = .07). To follow up these interactions, we conducted the paired samples *t* test. In the actor condition, there was no difference in discrimination accuracy between statements that were denied and statements that were described (*t* (57) = 0.60, *p* = .55, *d* = 0.08, 95% CI [− 0.18, 0.34]. In contrast, discrimination accuracy was significantly worse for statements that were denied (compared to description statements) when participants rated statements from speakers in the observer condition, *t* (57) = 2.70, *p* = .01, *d* = 0.36, 95% CI [0.09, 0.62]. The analysis of statement type × repetition revealed that for denials, participants did not differ in their ability to make accurate decisions as a function of whether the denial was stated once or thrice, *t* (115) = 0.52, *p* = .60, *d* = 0.05, 95% CI [− 0.13, 0.23]. However, for the description statements, participants were more accurate when asked to make judgments of descriptions that had been rehearsed once as opposed to descriptions that had been repeated three times, *t* (115) = 4.65, *p* < .01, *d* = 0.43, 95% CI [0.24, 0.62].

Finally, we observed a three-way interaction between role × statement type × repetition, *F* (1, 114) = 19.31, *p* < .01, η_p_^2^ = .15. To better understand this interaction, we conducted 2 × 2 (statement type × repetition) ANOVA separately for each role group. In the actor condition, there was only a significant main effect of repetition, *F* (1, 57) = 11.72, *p* < .01, η_p_^2^ = .17. Discrimination accuracy was significantly greater for statements that had been rehearsed only once compared to statements that had been rehearsed three times, *d* = 0.45, 95% CI [0.18, 0.72]. Neither the main effect of statement type (*F* (1, 57) = 1.81, *p* = .18, η_p_^2^ = .02) nor the interaction between statement type and role (*F* (1, 57) = 1.07, *p* = .31, η_p_^2^ = .02) were significant in the actor condition.

However, we see the opposite pattern in the observer condition: when participants rated statements provided by speakers describing actions they had witnessed, there was a main effect of statement type (*F* (1, 57) = 7.29, *p* = .01, η_p_^2^ = .11) and an interaction between statement type and repetition (*F* (1, 57) = 25.41, *p* < .01, η_p_^2^ = .31). We conducted the paired-samples *t* test to follow up on the two-way interaction. For once-rehearsed items, discrimination accuracy for denials was significantly worse than for descriptions (*d* = 0.73, 95% CI [0.44, 1.02]). However, there was no difference in discrimination accuracy for descriptions and denials when these statements had been rehearsed repeatedly (*d* = 0.25, 95% CI [− 0.01, 0.51]). In the observer condition, there was no main effect of repetition (*F* (1, 57) = 2.74, *p* = .10, η_p_^2^ = .05).

Given the small observed *d’* values, we further conducted the one-sample *t* test to determine which item types led to discrimination accuracy that was different from random chance performance. In the actor condition, only items that were denied once improved discrimination accuracy above chance performance (*t* (57) = 2.67, *p* = .01, *d* = 0.35, 95% CI [0.08, 0.61]. For the observer condition, participants were significantly greater than chance at discriminating between true and false statements that had been described once (*t* (57) = 3.21, *p* < .01, *d* = 0.42, 95% CI [0.15, 0.69]). Further, participants who viewed observer statements were worse than chance at discriminating true and false statements that were denied once (*t* (57) = 4.82, *p* < .01, *d* = 0.63, 95% CI [0.35, 0.91]) as well as repeatedly described (*t* (57) = 4.07, *p* < .01, *d* = 0.53, 95% CI [0.26, 0.81]).

#### Response bias

Next, we examine differences in response bias (*c*)*.* We conducted 2 (role: actor, observer) × 2 (statement type: deny, describe) × 2 (repetition: once, thrice) mixed factorial ANOVA on the response bias measure, with role serving as the between-subjects variable and statement type and repetition as the within-subjects variables. There was a significant main effect of repetition (*F* (1, 114) = 56.82, *p* < .01, η_p_^2^ = .33). Pairwise comparisons revealed that participants were more likely to judge repeatedly rehearsed statements as truthful than once rehearsed statements (*d* = 0.65, 95% CI [0.45, 0.85]). This is qualified by a significant two-way interaction between role and repetition (*F* (1, 114) = 20.95, *p* < .01, η_p_^2^ = .16). Follow-up comparisons revealed that there was a significantly greater tendency to judge repeated statements as truthful in the observer condition (*d* = 1.13, 95% CI [0.80, 1.46]) than in the actor condition (*d* = 0.27, 95% CI [0.01, 0.53]).

We further observed a significant interaction between role and statement type (*F* (1, 114) = 10.33, *p* < .01, η_p_^2^ = .08). Follow-up comparisons revealed that the pattern of response bias differed depending on whether the speaker was describing actions that had been performed or actions that had been observed. In the actor condition, participants were more likely to judge denied items (mean (M) = 0.19, SD = 0.51) as truthful than described items (M = − 0.09, SD = 0.44; *d* = 0.33, 95% CI [0.07, 0.60]). However, in the observer condition, participants were more likely to judge described items (M = 0.15, SD = 0.35) as truthful than denied items (M = 0.01, SD = 0.34; *d* = 0.26, 95% CI [0.00, 0.52]). The main effect of role (*F* (1, 114) = 0.40, *p* = .53, η_p_^2^ < .01), the main effect of statement type (*F* (1, 114) = 1.14, *p* = .29, η_p_^2^ = .01), the two-way interaction between statement type and repetition (*F* (1, 114) = 0.43, *p* = .51, η_p_^2^ < .01), and the three-way interaction between statement type, repetition, and role (*F* (1, 114) = 0.62, *p* = .43, η_p_^2^ = .01) were not statistically significant.

### Experiment 2

The analyses for experiment 2 were identical to those of experiment 1. The critical difference between experiments 1 and 2 is that we increased the number of video stimuli that were used in experiment 2.

#### Discrimination accuracy

We conducted 2 (role: actor, observer) × 2 (statement type: deny, describe) × 2 (repetition: once, thrice) mixed factorial ANOVA on the discrimination accuracy measure, with role serving as the between-subjects variable and statement type and repetition the within-subjects variables. Cell means and standard deviations can be found in Table 2. We observed significant main effects of statement type (*F* (1, 123) = 22.63, *p* < .01, η_p_^2^ = .16) and role (*F* (1, 123) = 11.12, *p* < .01, η_p_^2^ = .08). Discrimination accuracy was significantly worse for statements that were denied (compared to statements that were described; *d* = 0.43, 95% CI [0.24, 0.61]) and for statements where the speaker described actions they had observed (compared to statements about actions the speaker had performed themselves; *d* = 0.60, 95% CI [0.24, 0.95]). This was qualified by a significant interaction between statement type and repetition, *F* (1, 123) = 6.54, *p* = .01, η_p_^2^ = .05. There was no difference in discrimination accuracy for items denied once or three times (*d* = 0.05, 95% CI [− 0.12, 0.23]). In contrast, accuracy was greater for descriptions rehearsed once, rather than repeatedly (*d* = 0.27, 95% CI [0.09, 0.45]).

**Table 2:** Results in experiment 2

	Hit rate	False Alarm rate	*d’*	*c*
Actor				
Deny once	0.48 (0.17)	0.52 (0.15)	−0.13 (0.41)*	0.00 (0.46)
Deny thrice	0.48 (0.18)	0.48 (0.15)	−0.02 (0.42)	0.05 (0.43)
Describe once	0.53 (0.16)	0.46 (0.18)	0.21 (0. 43)*	0.02 (0.43)
Describe thrice	0.42 (0.17)	0.42 (0.19)	−0.01 (0.46)	0.23 (0.48)
Observer				
Deny once	0.38 (0.18)	0.47 (0.20)	−0.26 (0.58)*	0.22 (0.46)
Deny thrice	0.40 (0.21)	0.51 (0.19)	−0.31 (0.55)*	0.13 (0.54)
Describe once	0.37 (0.20)	0.36 (0.22)	0.06 (0.68)	0.40 (0.55)
Describe thrice	0.32 (0.19)	0.36 (0.21)	−0.11 (0.46)	0.46 (0.54)

Standard deviations are in parentheses. Values of *c* greater than 0 indicate a truth bias

*Represents values of *d’* that are significantly different from chance performance

Unlike experiment 1, the main effect of repetition did not reach the conventional level of significance (*F* (1, 123) = 3.72, *p* = .06, η_p_^2^ = .03). However, the same pattern from experiment 1 was observed. That is, accuracy for statements that were rehearsed repeatedly was marginally worse than for statements rehearsed once (*d* = 0.17, 95% CI [0.00, 0.35]). The two-way interactions between role and statement type (*F* (1, 123) = 0.89, *p* = .35, η_p_^2^ = .01), between role and repetition (*F* (1, 123) = 0.41, *p* =. 52, η_p_^2^ < .01), and the three-way interaction between role, statement type, and repetition (*F* (1, 123) = 1.35, *p* = .25, η_p_^2^ = .01) were not statistically significant.

We again conducted the one-sample *t* test to determine which item types led to discrimination accuracy that was different from random chance performance. In contrast to experiment 1, participants were worse than chance at discriminating items that were denied once by speakers in the actor condition, (*t* (61) = 2.48, *p* = .01, *d* = 0.32, 95% CI [0.06, 0.57], but were greater than chance at discriminating items that were described once (*t* (61) = 3.80, *p* < .01, *d* = 0.48, 95% CI [0.22, 0.74]). For the observer condition, participants were significantly *worse* than chance at discriminating between true and false statements that had been denied once (*t* (62) = 3.64, *p* < .01, *d* = 0.46, 95% CI [0.20, 0.72]) as well as repeatedly denied (*t* (62) = 4.39, *p* < .01, *d* = 0.55, 95% CI [0.29, 0.82]).

#### Response bias

We next conducted 2 (role: actor, observer) × 2 (statement type: deny, describe) × 2 (repetition: once, thrice) mixed factorial ANOVA on the response bias measure, with role serving as the between-subjects variable and statement type and repetition the within-subjects variables. We observed significant main effects of statement type (*F* (1, 123) = 7.41, *p* = .01, η_p_^2^ = .06), repetition (*F* (1, 123) = 7.59, *p* = .01, η_p_^2^ = .06), and role (*F* (1, 123) = 22.33, *p* < .01, η_p_^2^ = .15). Follow-up comparisons revealed that there was a greater tendency to judge descriptions as truthful than denials (*d* = 0.24, 95% CI [0.07, 0.42]) and repeated statements than once-rehearsed statements (*d* = 0.24, 95% CI [0.06, 0.41]) as being truthful. These main effects were qualified by a significant two-way interaction between statement type and repetition (*F* (1, 123) = 11.57, *p* < .01, η_p_^2^ = .09). Follow-up comparisons revealed there was no difference in response bias for denials that were rehearsed one time or three times (*t* (124) = 0.61, *p* = .54). However, there was a tendency to judge repeated descriptions as more truthful than once-rehearsed descriptions (*t* (124) = 4.24, *p* < .01, *d* = 0.38, 95% CI [0.20, 0.56]).

Further, there was a larger truth bias in the observer condition than in the actor condition (*d* = 0.85, 95% CI [0.48, 1.21]). This main effect of role was qualified by a significant two-way interaction between role and repetition (*F* (1, 123) = 10.55, *p* < .01, η_p_^2^ = .08). However, unlike in experiment 1, participants were more likely to judge repeated statements (rather than once-rehearsed statements) as truthful in the actor condition (*d* = 0.61, 95% CI [0.33, 0.87]). There was no difference in response bias for statements that were rehearsed once or three times in the observer condition (*d* = 0.04, 95% CI [− 0.21, 0.29]). The two-way interaction between role and statement type (*F* (1, 123) = 1.50, *p* = .22, η_p_^2^ = .01) and the three-way interaction between role, statement type, and repetition (*F* (1, 123) = 0.04, *p* = .84, η_p_^2^ < .01) were not significant.

### Discussion

The pattern of results was broadly similar across the two experiments, with the outcome of most analyses in experiment 2 directly replicating findings in experiment 1, or at least trending in the same direction. For some factors, there were inconsistencies between the two experiments, and this lack of replication should be considered when evaluating the implications of these results.

Across both experiments, we found consistent patterns for a main effect of role and repetition. Specifically, discrimination accuracy was significantly worse when participants evaluated speakers who had observed actions, rather than performed the actions themselves. Additionally, discrimination accuracy was significantly worse when statements were repeatedly practiced in experiment 1; though this effect was not statistically significant in experiment 2, the pattern was consistent.

Of particular interest is the statement type x repetition interaction that was replicated across experiments 1 and 2. Discrimination accuracy for denials did not differ as a function of the amount of repetition that statement received. Description statements, however, were more accurately identified as lies and truths when they had *not* been repeatedly practiced. In fact, practiced description statements often produced instances where repeated truthful statements were more likely to be perceived as deceptive than were actual deceptive statements. One potential reasoning for this finding is related to the presumption of innocence (Kassin, 2005). When asked to lie repeatedly, speakers in Dianiska et al. benefitted from being able to practice the content and delivery of their lie multiple times. Such practice may have reduced the amount of cognitive load in generating the lie (e.g., Vrij et al., 2009) and increased the fluidity of the statement. In contrast, when the speakers provided a truthful statement, perhaps they did not feel the need to put as much effort into being perceived as honest because they knew that they were telling the truth. Therefore, the statistically significant negative *d’* values observed in the current study could be due to differences in a speaker’s effort to appear truthful when providing deceptive statements.

## General discussion

The present studies offer evidence for the influence of rehearsal on deception detection. Through some theories of deception detection, (e.g., the cognitive load approach) it is reasonable to argue that having time to *rehearse* a lie would aid a deceiver in evading detection. Here, we experimentally demonstrate the consequences of that liar’s rehearsal. Repeated statements appeared to be a bit more difficult for people to identify as lies, as demonstrated by chance and worse-than-chance discrimination accuracy. Further, we introduced a novel paradigm to examine people’s ability to distinguish lies from truths about multiple statements from the *same* speaker (as opposed to typical studies wherein participants make judgments about a single statement from a speaker).

Taken as a whole, these data suggest that both deceptive and truthful descriptive statements may benefit from repetition, such that both are more convincing after having been repeated. This repetition could have been beneficial in increasing the fluency with which the speakers provided their account, suggesting that both initial truthful and deceptive statements are lacking this in the absence of repetition. This is an important notion that even if telling the truth is less cognitively demanding (DePaulo et al., 2003), doing so convincingly may require additional effort. As seen in previous research, the data reveal that lies are harder to detect when they have been repeated (Bond Jr & DePaulo, 2006). However, this finding is particularly important for the truthful statements, as it suggests that simply being truthful may not be enough to convincingly portray honesty to an individual who is attempting to assess that statement’s veracity. Rather, it seems that additional components from rehearsal also may benefit our ability to appear more truthful. Additionally, the random chance values that we observed on discrimination may also reflect the finding from the Bond Jr and DePaulo (2006) meta-analysis that people are at chance level when discriminating between truths and lies.

The data also suggest that differences in discerning truthful from deceptive statements may vary as a function of the type of information being reported. Across both studies, participants who viewed speakers describing self-performed actions (i.e., in the actor condition) were able to better discern between truthful and deceptive statements as opposed to the participants who viewed speakers describing actions performed by others (i.e., in the observer condition). This difference may arise due to participants being more sensitive to cues that can aid in detecting deception when hearing accounts from a first-person perspective rather than a third-person account. Alternatively, it may be the case that individuals are confronted more often with first-person lies as opposed to lies about a third party, making it easier for participants to parse between truthful and deceptive statements. A final interpretation of these data is that the statements in the actor and observer conditions differed in a way that allowed participants to more accurately assess first-person lies. The nature of this difference warrants future investigation to better understand this relationship.

These findings have important implications for lie detection. First, our data suggest that repetition may benefit both liars and truth-tellers. Given a truth teller’s presumption (or phenomenology) of innocence (Kassin, 2005), they may mistakenly believe that simply providing their account of an event will suffice and the truth will be just as compelling to other individuals. A liar, on the other hand, knows his account will be scrutinized and will likely have practiced his account multiple times, putting the truth-teller at a deficit that may make the truth-teller appear more deceptive than a liar (Granhag & Strömwall, 1999).

Second, we see throughout the study the difficulty in discriminating between true and false denials. Across both experiments, discrimination accuracy suffered when participants rated simple *denial* statements, compared to descriptions. Given that this task seems to be particularly cumbersome, it may benefit individuals tasked with assessing veracity to require interviewees to provide more detailed accounts (e.g., “I did not go to the store that night because I was at the cinema with Cheryl instead”), rather than accepting a denial-only statement. For instance, the use of a “model statement” in an interview has been shown to increase the length of both truthful and deceptive accounts (e.g., Leal, Vrij, Warmelink, Vernham, & Fisher, 2015). After providing an experimenter with an initial statement, both truthful and deceptive participants in Leal et al. (2015) were presented with an example statement that depicted the level of detail desired of participants and were then asked to provide their statements again. The use of an example statement led both liars and truth-tellers to add more details to their initial statements, yet overall detection accuracy was improved due to differences in the *plausibility* of the additional details. Thus, while true and false denials in the current study were difficult for raters to discriminate, it seems that requiring interviewees to provide more detailed statements (i.e., descriptions) offers raters more opportunities to detect lies.

Some limitations within the current study pertain to the nature of the videos that were utilized. In our study, we used more common, everyday lies. This is beneficial in providing an understanding of our ability to discriminate between truthful and deceptive statements in our daily life. However, it would also be beneficial to have a comparison between these low-stakes and high-stakes lies that are more commonly studied in the deception literature and are, arguably, more consequential in terms of the implications of accurate detection. Specifically, it is possible that a low-stakes lie may be less likely to be detected, as assessors of veracity may reason, “why would they bother to lie about that?” whereas the motivation for a high-stakes lie is often quite clear (e.g., to escape punishment). However, given that performance in the current set of studies was roughly similar to that in the study of Bond Jr and DePaulo (2006) in that accuracy hovered around chance, this may suggest that there are no differences between low-stakes and high-stakes lies. Additionally, the item types used in the current study were constrained to statement types that were used in the experiment by Dianiska et al. (in preparation). However, people lie in myriad ways that may not be captured by the statement types used here. For example, a liar may choose to provide an entirely novel account, or instead a liar could provide a *mostly* truthful account, changing only a few details. This lying strategy was not explored in the current study, but also warrants future investigation. Additionally, the fact that the speaker videos were presented in a predetermined order could have resulted in order effects.

An overarching finding reported in the literature on deception detection studies is how difficult it is for individuals to differentiate between truthful and deceptive statements. One approach that researchers have utilized to better discriminate between truthful and deceptive statements focus on examining the *content* of a statement, through tools like Criteria-Based Content Analysis (CBCA; Berliner & Conte, 1993; Steller & Kohnken, 1989). Further, the Assessment Criteria Indicative of Deception (ACID; Colwell, Hiscock-Anisman, Memon, Taylor, & Prewett, 2007) and Psychologically-Based Credibility Assessment Tool (PBCAT; Evans & Michael, 2014; Evans et al., 2013) have also been used to help assessors of veracity make more accurate judgments. It remains to be seen if these would aid in discriminating between the repeated statements used in the current study.

The cognitive-load approach has also been shown to be beneficial with regard to differentiating between truthful and deceptive statements. Because lying is more cognitively demanding than telling the truth, an interview approach that exploits this difference (e.g., recalling an event in reverse order; Evans et al., 2013) can aid in discrimination. However, it remains to be seen if these benefits would still be observed when a liar had time to practice his or her statement. Given that most liars know their narratives will be met with skepticism and they thus prepare a practiced and elaborate account accordingly, these findings highlight a danger for truth-tellers who may not feel the need to practice their account and, therefore, it may be less compelling to evaluators.

## Conclusion

Differentiating between truthful and deceptive statements has been shown to be a daunting task that individuals struggle to accurately perform. To add to the complexity, factors such as statement type and repetition drastically impact our ability to make accurate veracity assessments. In the current studies, we showed that discrimination between lies and truths is most effective when evaluating more elaborated statements (i.e., descriptions), and when evaluating statements that had not been repeatedly repeated. Though deception detection research often focuses on accurate identification of a liar, in some settings it is just as important to be able to recognize a truthful statement. Compared to an individual providing a lie, a truth-teller may be less likely to be believed should they provide an unrepeated account. These findings expand on our current knowledge of deception detection and add to the literature by further identifying instances where deception detection may be impaired.

## Data Availability

The data sets analyzed during the current study are available on the OSF.

## Appendix

**Table 3:** Accuracy for all item types in experiments 1 and 2

		Describe		Deny	
		Once	Thrice	Once	Thrice
Experiment 1					
Actor	Lie	0.56 (.17)	0.5 (.19)	0.47 (.22)	0.41 (.22)
	Truth	0.47 (.18)	0.47 (.21)	0.59 (.21)	0.54 (.21)
Observer	Lie	0.53 (.20)	0.36 (.13)	0.49 (.14)	0.44 (.16)
	Truth	0.54 (.15)	0.56 (.15)	0.4 (.14)	0.54 (.19)
Experiment 2					
Actor	Lie	0.53 (.16)	0.42 (.18)	0.47 (.18)	0.48 (.19)
	Truth	0.55 (.18)	0.58 (.20)	0.48 (.15)	0.52 (.15)
Observer	Lie	0.37 (.20)	0.32 (.19)	0.38 (.18)	0.39 (.22)
	Truth	0.63 (.24)	0.65 (.21)	0.51 (.20)	0.49 (.19)

Note. Standard deviations are in parentheses
